# Annual Biochar Application Regulates Maize Internode Development and Yield by Modulating Photosystem II Photosynthetic Efficiency

**DOI:** 10.3390/plants15081141

**Published:** 2026-04-08

**Authors:** Yanghui Sui, Jiping Gao, Dawei Wang, Yang Zhang, Yusheng Ye, Wanxin Xiao, Yanbo Wang

**Affiliations:** 1 Corn Research Institute, Liaoning Academy of Agricultural Sciences, Shenyang 110161, China; davidww2000@126.com (D.W.); zy52789@126.com (Y.Z.); yysh05@126.com (Y.Y.); xiaowanxin2011@126.com (W.X.); lnwangyanbo@163.com (Y.W.); 2 Rice Research Institute, Agronomy College, Shenyang Agricultural University, Shenyang 110866, China; jipinggao@syau.edu.cn

**Keywords:** biochar, internode development, spring maize, fluorescence parameters, yields

## Abstract

Excessive planting density and heavy rainfall weather threatens global agriculture, particularly affecting maize. Biochar is an environmentally friendly soil amendment that has a yield-increasing effect. However, the regulatory mechanism of biochar frequency on crop internode development and photosystem II photosynthetic efficiency remains unknown. A total of nine treatments were followed in this experiment. Three applications of biochar were as follows: no biochar application (B0); biochar application at 4.2 t ha^−1^ year^−1^ (B1); and biochar application at 8.4 t ha^−1^ 2 year^−1^ (B2), alongside three nitrogen (N) fertilizer rates (0, N0; 180 kg ha^−1^, N1 and 225 kg ha^−1^, N2). The results showed that the internode thickness of the 2nd to 5th nodes under N2B2 treatment increased by 17.7%, 16.0%, 19.7%, and 21.7%, respectively, compared to N0B0. Annual biochar application had a higher stem diameter coefficient for the 1st to 3rd nodes than no biochar (B0) and treatments applied every two years (B2). Annual biochar application had the highest dry weight of internodes and plant height compared with B0 and B2. The relative chlorophyll content of leaves was significantly increased by biochar combined with N fertilizer or by N fertilizer alone. Biochar combined with N fertilizer significantly reduced NPQt and ΦNPQ, which were reduced by 59% and 50%, respectively, under N2B1 treatment compared with N0B0. The N2B1 treatment increased ΦII by 30% compared to N0B0. Stem diameter coefficient was significantly negatively correlated with NPQt and ΦNPQ and significantly positively correlated with ΦII and Fv/Fm. Compared to B1, B2 increased the maize yield. Annual biochar application combined with N fertilizer reduced stem collapse and enhanced post-flowering photosynthesis. Overall, considering the yield traits, 8.4 t ha^−1^ biochar application combined with 180 kg ha^−1^ N fertilizer treatment was the best. This study will provide reference data for cultivation regulation to enhance maize’s resistance to collapse and maintain photosynthetic capacity.

## 1. Introduction

In cereal crops such as maize, which is one of the most widely cultivated, especially for high-yielding populations, lodging largely impairs grain yield and efficient mechanical harvesting [1,2]. Modern high-yield production, including high-yield breeding and high-yield cultivation techniques, is mainly achieved through large-panicle varieties, high planting density, and heavy fertilizer input [3]. This leads to reducing stem strength and increasing lodging risk [4] when heavy rain occurs or the strong winds blow [5,6].

An effective way to address these problems is to enhance stem strength [7,8,9]. Some cultivation measures, such as reducing planting density, decreasing N fertilizer inputting, and using growth regulators, have tried to strengthen maize stalk but had significant drawbacks, which limited the yield improvement [10]. So, it is necessary to find a method that can not only promote the crop production capacity but also enhance the stem strength.

Biochar, as an effective soil amendment, has been widely used in agricultural fields [11,12,13]. There are generally two methods for applying biochar: one is to apply it continuously every growing season [14,15], and the other is to add a large amount at one time, equivalent to the dosage for several growing seasons or years [16,17]. Some research on the effect of biochar on crop stem growth has focused on rice. The major findings indicated that biochar enhances lodging resistance by increasing stem wall thickness and plumpness. Due to its ability to hold nutrients, biochar released NH_4_^+^ as the sole N source resulted in thickened cell walls. Additionally, biochar generates a more negative electrical potential at the root epidermal cell layer, driving Si uptake [18]. Gong et al. [19] found that 5–10 t ha^−1^ biochar can reduce the lodging index by increasing the number of large and small vascular bundles and by raising lignin and silicon content in rice stems. Previous studies mainly focused on the lodging characteristics at different internodes. Stalk lodging mainly occurs in the 2nd to 5th internodes [20]. Changes in the dimensions of the stalk cross-section were more sensitive to external stresses than changes in the material properties of the stalk internode [21]. The stalk cross-section is highly predictive of stalk strength, and it appears to be largely unaffected by hybridization, planting area, or planting density [22].

Chlorophyll fluorescence parameters have been confirmed to play important roles in providing early warning of crop abiotic stress [23,24]. Previous studies on chlorophyll fluorescence have concentrated on various stress conditions [25]. For example, He et al. [26] found that low oxygen stress caused by waterlogging may destroy the photosynthetic electron transport chain of PSII. Similarly, Ratajczak et al. [27] examined the recovery capability of phytostimulators on chlorophyll fluorescence and photosynthetic parameters under cold stress. Twenty days after using the phytostimulator, the effective quantum yield of photosystem II, maximum photosynthetic efficiency of PSII, and electron transport rate of maize leaves were increased, indicating recovery from cold stress. In actuality, it is common to find that nitrogen fertilizer improves the performance of photosynthetic system and the efficiency of electron transfer [28]. Likewise, Wu et al. [29] reported that 48 t ha^−1^ biochar enhanced the potential photochemical efficiency of the PSII reaction center and produced the greatest increase in soybean yield the. Similarly, when biochar was added to the paddy soil, the application of 8 t ha^−1^ biochar increased the light energy conversion efficiency of the PSII reaction center [30].

Current studies on biochar have concentrated on crop yield. For example, Yu et al. [31] examined the incorporation of biochar with dicyandiamide and its effects on soybean–wheat yield. They found that the application of 30 t ha^−1^ biochar increased the annual total output of soybeans and wheat by more than 15%. Some studies have found that biochar combined with organic fertilizer improves root development and increases rice yield [32,33]. This is because it improves the availability of nutrients, regulates the C/N balance, and leads to an increase in the utilization efficiency of soil nitrogen [34]. It is presumed that the effect of biochar on stem characteristics is mainly regulated by PSII fluorescence. Therefore, system research of the effects of a biennial application of biochar on the fluorescence characteristics and stem resistance traits of maize is a worthy topic, which could offer new insights into the mechanistic coupling between photosynthetic performance and stem properties in response to biochar.

Herein, this study uses two-year/annual application of biochar under a 6-year experiment, and the following hypothesis was proposed: biochar amendment improves stem properties by enhancing PSII efficiency during the post-flowering stage. The results of this study are expected to provide theoretical support for the scientific evaluation of the long-term effects of biochar.

## 2. Results

### 2.1. Effects of Biochar Amendment on Agronomic Traits of Maize Stems

Five years after biochar application, both the length of the 1st to 5th internode and stem diameter of maize increased (Table 1). All treatments affected internode the lengths of the 2nd to 5th internodes, except that of the 1st internode. In the N0 level, the mean length of B2 treatment was the highest, which was 8.58 cm. Only biochar treatments, the B1 treatment exhibited the greatest average diameter of at 16.92 mm, while the B2 treatment exhibited the smallest average diameter of 16.43 mm. In the N1 level, the average length of the 1st to 5th internodes increased with increasing biochar amount, while the stem diameter decreased. At the N2 level, the B1 treatment had the highest average internode length at 10.83 cm, and B2 treatment showed the lowest at 9.37 cm. The biochar only treatment had a significant impact on the 5th internode length (*p* < 0.05). The interaction between biochar and nitrogen fertilizer on stem thickness was significant between the 2nd and 4th internodes. The length of the 4th internode was significantly different between the N2B1 and N0B0 treatments (*p* < 0.05), as well as between the N2B2 and N0B0 treatments. Similar differences were observed for the 5th internode. Biochar did not influence the length and diameter of the 1st to 4th internodes, but nitrogen fertilization significantly influenced the 2nd to 5th internodes (all *p* < 0.05). Significant interactions were found for length (*p* < 0.01) and diameter (*p* < 0.05) of the 4th internode.

Biochar did not significantly influence the stalk width coefficient and the moment of inertia of the 1st–5th internodes, but it enhanced the moment of inertia of the 4th and 5th internodes by 4.3% and 4.4% (B1), 6.5%, and 2.6% (B2), respectively (Table 2). We find that nitrogen fertilization significantly influenced the stalk width coefficient and the moment of inertia of the 3rd to 5th internodes.

In Table 3, different biochar treatments had different effects on internode dry weight and plant height. First, the B1 treatment has the greatest improvement in 3rd to 5th internodes, with dry weights of 5.16, 5.53, and 5.40 g, representing the increases of 7.3, 13.6, and 17.9% compared to the B0 treatment. However, the effect was not significant (*p* > 0.05). The B1 treatment resulted in the most significant increase in plant height, reaching 266.63 cm. The plant height of the B1 and B2 treatments increased by 6.2 and 3.0%, respectively, compared with B0. In terms of internode dry weight and plant height, the biochar influence was weaker than that of N fertilization. Statistically significant influence was observed in N fertilization. Significant interactions of biochar and N fertilization were found for stem node dry weight and plant height except for 1st inernode (all *p* < 0.05 or *p* < 0.01).

### 2.2. Chlorophyll a Fluorescence Measurements Under Biochar Treatments

**Figure 1:**
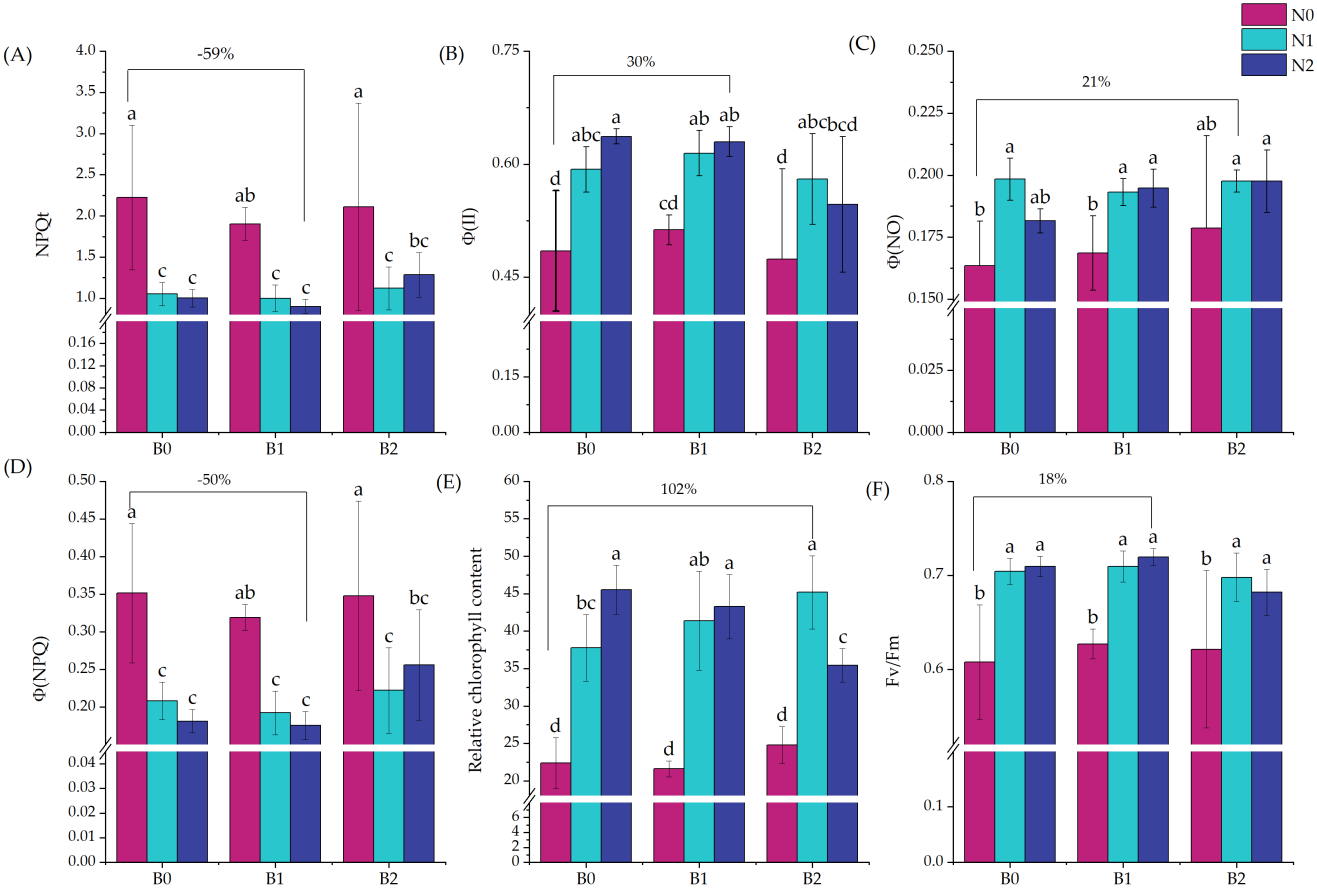
Effect of different biochar treatments on chlorophyll a fluorescence measurements of maize ear leaf. (**A**) NPQt, kinetics for induction of non-photochemical quenching; (**B**) Φ(II), PSII quantum yield; (**C**) Φ(NO), fraction of energy absorbed by PSII that is lost to unregulated processes; (**D**) Φ(NPQ), NPQ quantum yield; (**E**) relative chlorophyll content, and (**F**) Fv/Fm, the maximal quantum yield of PSII photochemistry. Different letters above the error bars indicate significant differences (*p* < 0.05) among the treatments. N0—Nil nitrogen; N1—180 kg Nha^−1^; N2—225 kg Nha^−1^; B0—Nil biochar; B1—4.2 t ha^−1^ year^−1^ of biochar; B2—8.4 t ha^−1^ 2 year^−1^ of biochar.

The NPQt measurements for the nine treatments revealed that maize under biochar treatments differed from N fertilization (Figure 1A). NPQt was decreased sharply after the N fertilization. The NPQt value ranged from 0.90 to 2.22. Furthermore, NPQ tension was associated with a rise in Φ(II) (PSII quantum yield). No differences in biochar treatments were observed in Figure 1A. Biochar and N fertilizer led to a substantial reduction in NPQt, with the N2B1 treatment exhibiting a significantly lower value compared to the N0B0 treatment, indicating a decrease of approximately 59%.

Φ(II) significantly changed (*p* < 0.05) in N fertilization. The incorporation of biochar and N fertilizer resulted in a substantial enhancement of Φ(II). Under the N2B1 treatment, Φ(II) significantly (*p* < 0.05) improved, by approximately 30%, compared to the N0B0 treatment. However, Φ(II) significantly declined due to the treatment with N2B2, while no change was observed for the treatment with N2B1 compared to N2B0. This finding suggests that the concurrent application of biochar and nitrogen fertilizer can enhance the photochemical efficiency of PSII. The corresponding values of Φ(NO) and Φ(NPQ) are given in Figure 1. Under both B1 and B2 treatments, no significant change in photosynthetic efficiency was observed. N fertilization improved Φ(NO) but reduced Φ(NPQ). Φ(NO) of the N1B2 treatment was increased by approximately 21% compared to N0B0. Meanwhile, the maximum of Φ(NPQ) was reduced by 50% under N2B1. These data indicate that moderate biochar combined with N fertilizer stimulates the PSII antenna system to capture more photosynthetically active radiation (PAR) efficiently.

Under the N0 conditions, no significant differences were observed in the Chl content and Fv/Fm parameter (Figure 1F). Fv/Fm ranged from 0.61 to 0.72 during the flowering stage. Both biochar combined with N fertilizer and N fertilizer alone significantly increased the Fv/Fm ratio and Chl content. The N2B1 treatment showed a higher Fv/Fm than the N0B0, with an increase of approximately 18%.

In Table 4, maize yield differed among the treatments. The yield of the B0 treatment was the highest yield compared with those of the B2 and B1 treatments, but the difference was not significant. The highest yield (12,977 kg/ha) was achieved under the N2B0 treatment, with increases of 36.4% and 21.7%, compared to the N2B2 and N1B2 treatments, respectively. The 1000-grain weight of the B2 treatment significantly increased by 3.5 and 3.2%, respectively, compared with the B0 and B1 treatments. However, the B2 treatment significantly reduced the number of kernels per ear (*p* < 0.05). Biochar and its interaction with N significantly influenced 1000-grain weight and kernels per ear. This indicates that N fertilizer exerts the most significant influence on yield when it comes to biochar treatments.

**Figure 2:**
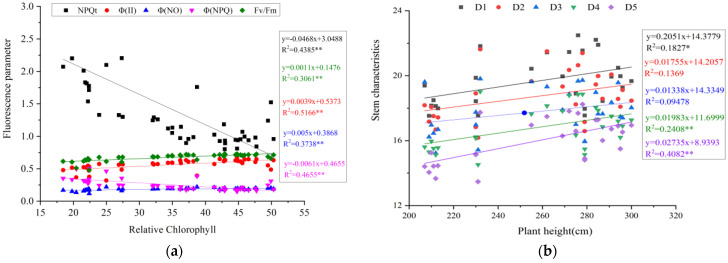
Correlation between chlorophyll content and fluorescence parameters (**a**) and plant height and stem thickness (**b**) under different biochar treatments. D1—1st internode; D2—2nd internode; D3—3rd internode; D4—4th internode; D5—5th internode. Stars indicate the level of significance (* *p* < 0.05, ** *p* < 0.01). The different colored lines represent the linear fitting of the corresponding legends.

The analysis of the correlation between relative chlorophyll content and fluorescence parameters revealed (Figure 2a) that during the flowering period, chlorophyll content was significantly negatively correlated with NPQt and Φ(NPQ) (*p* < 0.01) while exhibiting a significant positive correlation with Φ(II), Φ(NO), and Fv/Fm (*p* < 0.01). When it comes to the correlation between plant height and stem thickness (Figure 2b), there was a significant positive correlation at the 4th and 5th internodes (*p* < 0.01) and a significant positive correlation with stem diameter at the 1st internode (*p* < 0.05).

**Figure 3:**
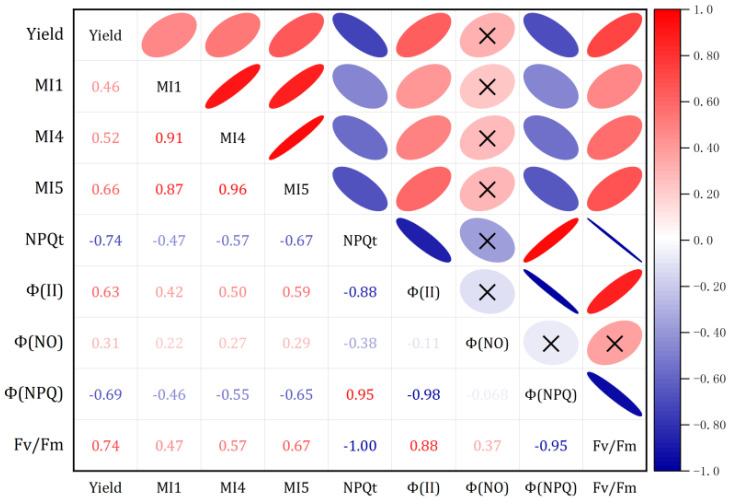
The correlation of main internode characteristics, fluorescence parameters, and yield. MI1—cross-sectional moment of inertia of 1st internode; MI4—cross-sectional moment of inertia of 4th internode; MI5—cross-sectional moment of inertia of 5th internode. The red oval indicate positive correlation; The blue oval indicate negative correlation; “X” indicate no significant correlation.

The correlation analysis indicated that, under the co-application of biochar and N fertilizer, maize yield was significantly negatively correlated with NPQt and Φ(NPQ), but it showed a significant positive correlation with Φ(II) and Fv/Fm (Figure 3). The cross-sectional moment of inertia of the 1st internode exhibits a significant negative correlation with NPQt and Φ(NPQ), as well as a significant positive correlation with Φ(II) and Fv/Fm. However, the sectional moment of inertia of the 4th and 5th internodes showed stronger correlations with fluorescence parameters.

## 3. Discussion

Current research often overlooks the long-term effectiveness of biochar in enhancing internode development and instead assumes that a one-time application, such as B2 treatment in this study, is sufficient. A 6-year field experiment indicated that the N2B2 treatment enhanced the thickness of the 4th and 5th internodes, as well as their cross-sectional moment of inertia. Luckily, our research further demonstrated that the nitrogen–biochar interaction occurred only on 4th and 5th internodes. After 6 years, the nitrogen–biochar interaction on yield still persisted. This is mainly attributed to nitrogen–biochar interaction’s sustained leaf photosynthetic efficiency in maize over the years.

### 3.1. Effects of Different Applications of Biochar on Stem Characteristics and Mazie Yield

Although biochar addition alone had no significant effect on the lodging resistance of maize after 6 years, the combined application of N fertilizer and biochar enhanced the lodging resistance of maize, especially at 180–225 kg N ha^−1^ (Table 1 and Table 2). The reason for this was that the internode thickness significantly increased, and the moment of inertia further increased (Table 1 and Table 2). Biochar can enhance crop nutrient uptake [35] and promote dry matter accumulation [36], thereby increasing crop yield [37]. Our experiment indicates that biochar combined with reduced N increased internode lengths of the 2nd to 5th nodes (Table 1) while decreasing cross-sectional moment of inertia and reducing lodging resistance. In contrast, biochar combined with conventional N (equal to 225 kg N ha^−1^) significantly increased the stem thickness of the 2nd to 5th inernodes and enhanced lodging resistance, which is consistent with previous findings [18,19]. This study also discovered that biochar combined with reduced N decreased the sectional moment of inertia of the 2nd to 5th internodes and that the 5th section had the smallest decrease. However, when combined with conventional N fertilizer, the sectional moment of inertia of the 2nd to 5th internodes increased, with the 5th section showing the smallest increase. This indicated that 5th internode is not sensitive to N fertilizer. Compared with the B0 and B2 treatments, the sectional moment of inertia of the 1st–5th internodes in the B1 treatment increased, except at the 4th internode (Table 2), indicating that continuous application of biochar can reduce the risk of stem lodging. Gong et al. [19] found that biochar enhances rice lodging resistance by reducing the lengths of the 1st and 2nd internodes while increasing inner and outer diameters, which is consistent with our findings. In addition, further research demonstrated that biochar improves rice mechanical strength by promoting silica, hemicellulose, and lignin deposition in stem cell walls [18]. The effect of adding biochar to different crops is different. In this study, the application rates of 4.2–8.4 t ha^−1^ have been consistently shown to improve photosynthetic performance and stem strength. But in rice, the effective biochar application rate was 30 t ha^−1^, derived from rice husk, which enhanced rice biomass and lodging resistance [18]. Previous studies have shown that straw-derived and wood-derived biochars can effectively reduce the lodging index [34]. This physiological basis may be attributed to the cell wall structure. Ammonium and nitrate are the primary nitrogen sources for key cell wall polymers such as lignin, hemicellulose, and cellulose, and the deposition of these polymers directly affects stem bending resistance [18,38]. Additionally, differences in resistance parameters among biochars derived from various feedstocks may be related to specific elements they contain. For instance, husk-derived biochar is rich in silicon [39], and the transport and deposition of silicon in plants can significantly increase stem wall thickness, thereby further enhancing lodging resistance. Excessive N fertilization could bring about more weight to the basal stems and higher plant height, making the crops increase the risk of lodging [40]. We observed that grain yield under the co-application of N and biochar was slightly lower compared with the high-N treatment (Table 4), but it was more beneficial for crop height (Table 3), and it reduced lodging risk (Table 2). Therefore, biochar can coordinate the contradiction between high yield and lodging risk under high-N fertilizer conditions [41].

### 3.2. Effects of Different Applications of Biochar on Photosynthesis

Chlorophyll fluorescence is a crucial parameter for photosynthesis, reflecting the absorption and utilization of light energy by PSII. Previous studies indicated that 10 t ha^−1^ biochar enhanced peanut leaf photosynthetic capacity [42], primarily by increasing chlorophyll fluorescence parameters, including photochemical quenching, actual photochemical efficiency, and net photosynthetic rate. Our study also established that the interactions between biochar and N fertilization have significantly improved ΦII. El-Desouki et al. [43] reported that the combined application of biochar and P fertilizer enhanced the maximum photochemical quantum yield of PSII (Fv/Fm) while also significantly increasing the electron transport rate and photosynthetic rate. In our study, both Fv/Fm and ΦNO were significantly enhanced under the combination of biochar and N fertilizer, as well as under N fertilizer alone. This suggests that the combination of biochar and N fertilizer enhances the efficiency of light quantum capture by the PSII reaction center, thereby mitigating the suppression of photosynthesis caused by environmental stress [44]. The different raw materials, carbonization temperatures, and application amounts of biochar have shaped crop responses, ultimately leading to the observed results. Similarly, 10 t ha^−1^ biochar significantly reduced ΦNPQ and ΦNO during peanut pod development, while 40 t ha^−1^ biochar increased ΦNPQ [42], which partially contradicts with our findings. These results suggest that light energy absorbed by PSII antenna pigments may be dissipated as heat rather than transferred through photochemical electron transport [45]. Only a few studies reported that biochar produced under 600 °C reduced NPQ in salt-alkali-tolerant ramie [46], which is consistent with our findings. We recorded that the co-application of biochar and N fertilizer significantly reduced NPQ in maize. This influence was largely affected by enhancing the relative chlorophyll content of maize [47], thereby ensuring the synthesis of various enzymes and electron carriers involved in photosynthetic carbon assimilation, ultimately improving leaf photosynthetic function [48]. Consequently, more light energy is absorbed, resulting in a reduced NPQ. Additionally, biochar may promote root morphology development [49], which enables greater nutrient and water uptake, thereby enhancing photosynthetic performance [50]. In our previous study, we reported that applying 8.4 t ha^−1^ biochar promotes fine root growth of maize [51]. Considering the correlations among these indicators, long-term biochar application enhances the relationship between agronomic traits at the first, fourth, and fifth internodes, as well as the fluorescence characteristics of leaves. Our findings are consistent with those of Kong et al. [52], who reported that lodging resistance in densely planted maize depends most strongly on the mechanical properties of the 3rd to 5th internodes. Similarly, Xue et al. [53] demonstrated that basal internodes showed the greatest plasticity in response to agronomic measures. Therefore, this study infers that the basal internodes are also sensitive to photosynthetic efficiency. This suggests that biochar’s modification of internode traits may be linked to leaf photosynthesis.

Although this study has achieved certain results in exploring the impact of N and alternate-year application of biochar on the internodes of maize, there were still some limitations. We have explored the effect of the fluorescence properties, especially the photosystem II (PSII) reaction center. However, future studies can delve into the microscopic characteristics of stems and substance transport.

## 4. Materials and Methods

### 4.1. Site Description

**Figure 4:**
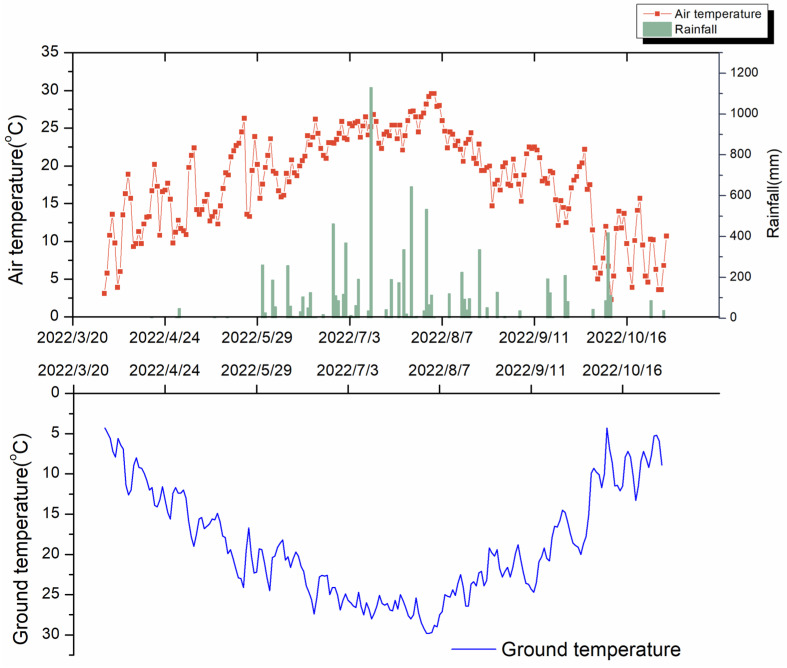
Mean daily air temperature (°C), precipitation (mm), and ground temperature in 2022.

The experiment was conducted at the Liaoning Academy of Agricultural Sciences (42.0344° N, 123.5698° E), Liaoning Province, China. The climate in this region is classified as the northern temperate continental monsoon climate. Figure 4 shows the climate variables observed during the growing season (March to October). This region is mainly characterized by continuous maize cropping, and the soil is classified as Udalfs according to the USDA Soil Taxonomy, which corresponds to brown soil in the Chinese Genetic Soil Classification. The basic properties of the soil are as follows: organic matter of 14.1 g kg^−1^, alkaline hydrolysis nitrogen of 94.7 mg kg^−1^, total nitrogen (TN) of 1.08 g kg^−1^, total phosphorus (TP) of 0.52 g kg^−1^, total potassium of 18.05 g kg^−1^, available phosphorus (AP) of 27.2 mg kg^−1^, and available potassium (AK) of 79 mg kg^−1^; pH = 6.3 [51].

The maize cultivar used was Zhengdan 958, and different amounts of biochar were applied to the soil in 2017 as a regulator to assess its long-term effects on maize. Maize straw was utilized as feedstock, and biochar was produced by Jilin Hongyuan Jialianhe Biomass Energy Co., Ltd., Jilin, China The biochar preparation process follows the parameters described by Gao et al. [54]. The properties of biochar are as follows: total carbon and nitrogen contents were 46.6% and 1.21%, respectively, and the pH value was 9.2. Biochar was uniformly applied to the upper soil surface of each plot before sowing, and it was evenly spun into the soil at a depth of about 10 cm. All treatments were normally applied with P and K fertilizers. The dosages of phosphorus and potassium were 120 kg P_2_O_5_ ha^−1^ and 90 kg K_2_O ha^−1^, respectively. All nitrogen, phosphorus, and potassium fertilizers should be applied to the soil at one time as base fertilizers during maize sowing. The planting density in all treatments was 67,500 plants ha^−1^, with sowing carried out in late April every spring. For each replicate, each treatment was carried out in a 6 m long 16-row plot, with a 0.6 m inter-row spacing.

### 4.2. Experimental Design

The experiment was conducted using a completely randomized plot design (Table 5). The trials were conducted in nine experimental blocks, comprising three biochar management treatments with triplicate replication: B0, no biochar application; B1, biochar application at 4.2 t ha^−1^ year^−1^; and B2, biochar application at 8.4 t ha^−1^ 2 year^−1^. In the 6-year field experiment, the experimental area and agricultural management were consistent across all treatments. Additionally, the total amount of biochar incorporation was the same for the B1 and B2 scenarios. The three nitrogen treatments included no N addition (N0), 20% reduction in N at 180 kg ha^−1^ year^−1^ (N1), and conventional N fertilizer application at 225 kg ha^−1^ year^−1^ (N2).

### 4.3. Field Investigation and Measurement

The following traits were measured in the field experiment: length and thickness of the 1st to 5th internodes, plant height, grain yield, and yield components.

Plant height was measured during the silking stage (R1) for 10 plants per plot in each of the three replicate blocks, resulting in a total of 30 plants measured per treatment.

#### 4.3.1. Stalk Agronomic Traits

Five representative plants of each block from each replicate that were as close as possible to the calculated mean plant height were used for the measurement of the internode. The internode length (IL) and internode diameter (ID) traits of the stalk were measured using electronic micrometers. Based on the measured indicator values, we calculated the cross-sectional moment of inertia (CSM, Equation (1)) and the stem diameter coefficient (SDC, Equation (2)).

(1) CSM = πd^4^/64,

(2) SDC = IL/ID,

#### 4.3.2. Analysis of Chlorophyll Fluorescence

An ultra-portable MultispeQ device (PhotosynQ, East Lansing, MI, USA) connected to the PhotosynQ platform (www.photosynq.org, accessed on 11 August 2022) was used to determine the maximal quantum yield of PSII photochemistry (Fv/Fm), quantum yield of PSII photochemistry (ΦII), quantum yield of non-photochemical energy loss [Φ(NPQ)], kinetics for induction of non-photochemical quenching (NPQt), and fraction of energy absorbed by PSII that is lost to unregulated processes [Φ(NO)]. Chlorophyll (Chl) fluorescence parameters were measured in a field from 9:00 to 11:30 a.m. Weather conditions were normal during the investigation. The ear leaf of three randomly selected plants per treatment was clamped in the leaf chamber to measure chlorophyll fluorescence. The measurements were taken from the middle part of the leaf.

#### 4.3.3. Grain Yield

At the R6 stage in October, the plants were harvested from 12-m^2^ areas comprising two middle rows within each block for the determination of grain yield, which was adjusted to 14% moisture content [55]. A total of 10 standard ears were selected, which were the closest to the mean from each plot, and row numbers and kernels per row of each ear was counted.

### 4.4. Statistical Analysis

The differences between treatment means were analyzed using SPSS statistics 17.0 (IBM, NY, USA) using two-way ANOVA (Duncan’s method, *p* < 0.05). Plotting was performed using OriginPro 8.5.

## 5. Conclusions

Our findings showed that annual biochar application combined with N fertilizer significantly increased the internode diameter of the 2nd to 5th internodes. Biochar combined with N fertilizer significantly enhanced maize photosynthetic capacity after flowering, ensuring post-flowering photochemical electron transport and reducing dissipation in the form of heat. The sectional moment of inertia of the 4th and 5th internodes showed stronger correlations with fluorescence parameters. Future research should further investigate how the synergistic effects of biochar modulate crop growth across different soil types and crops, as well as optimize biochar application strategies to enhance crop yield and resilience.

## Figures and Tables

**Table 1:** Changes in maize plant height, internode length, and thickness from 1st to 5th internodes under different biochar treatments.

Treatment		1st		2nd		3rd		4th		5th	
		Length/cm	Diameter/mm	Length/cm	Diameter/mm	Length/cm	Diameter/mm	Length/cm	Diameter/mm	Length/cm	Diameter/mm
B0	N0	5.57 ± 0.23 a	18.00 ± 0.48 b	7.43 ± 0.75 cd	17.37 ± 0.18 cd	8.70 ± 0.26 c	16.46 ± 0.23 c	9.23 ± 0.61 c	15.46 ± 0.14 c	10.80 ± 0.50 d	14.31 ± 0.23 b
	N1	8.27 ± 2.24 a	21.23 ± 0.72 a	8.13 ± 0.49 bcd	20.93 ± 1.10 a	11.23 ± 0.72 abc	19.59 ± 0.25 a	13.87 ± 0.51 b	18.27 ± 0.70 a	14.20 ± 0.66 c	17.40 ± 0.40 a
	N2	7.50 ± 0.75 a	19.55 ± 0.84 ab	9.70 ± 0.20 ab	18.64 ± 0.45 bcd	12.13 ± 0.29 ab	17.99 ± 1.38 abc	15.80 ± 0.87 a	16.81 ± 0.44 abc	16.90 ± 0.92 ab	16.67 ± 0.20 a
Average		7.11 ± 1.69 A	19.59 ± 1.52 A	8.42 ± 1.11 A	18.98 ± 1.67 A	10.69 ± 1.60 A	18.01 ± 1.53 A	12.97 ± 2.98 A	16.85 ± 1.29 A	13.97 ± 2.72 B	16.13 ± 1.42 A
B1	N0	4.43 ± 0.93 a	19.13 ± 0.90 ab	6.57 ± 1.07 d	17.92 ± 1.14 cd	8.93 ± 0.38 bc	17.53 ± 2.20 abc	10.10 ± 0.56 c	15.63 ± 0.51 c	10.93 ± 0.50 d	14.41 ± 0.74 b
	N1	5.73 ± 2.35 a	20.85 ± 2.09 a	8.63 ± 1.10 abc	19.34 ± 0.71 abc	12.90 ± 3.50 a	18.09 ± 0.76 abc	13.17 ± 1.51 b	17.59 ± 0.65 ab	15.47 ± 1.08 bc	16.86 ± 0.74 a
	N2	7.60 ± 2.48 a	19.86 ± 0.17 ab	10.60 ± 0.53 a	18.87 ± 1.05 abcd	11.70 ± 2.05 abc	17.95 ± 0.45 abc	16.43 ± 1.19 a	16.99 ± 0.70 abc	18.20 ± 0.26 a	16.39 ± 0.78 a
Average		5.92 ± 2.24 A	19.95 ± 1.36 A	8.60 ± 1.93 A	18.71 ± 1.06 A	11.18 ± 2.69 A	17.86 ± 1.21 A	13.23 ± 2.92 A	16.74 ± 1.02 A	14.87 ± 3.24 A	15.89 ± 1.30 A
B2	N0	5.93 ± 3.33 a	18.05 ± 0.45 b	7.53 ± 2.15 cd	17.03 ± 0.96 d	9.03 ± 1.42 bc	16.45 ± 0.89 c	9.80 ± 0.75 c	15.57 ± 0.92 c	11.13 ± 0.67 d	14.63 ± 1.01 b
	N1	8.93 ± 4.91 a	18.99 ± 2.15 ab	8.90 ± 1.06 abc	18.03 ± 2.03 cd	13.27 ± 1.88 a	16.92 ± 1.68 bc	16.00 ± 1.08 a	16.47 ± 2.02 ab	18.00 ± 1.45 a	15.84 ± 1.71 ab
	N2	6.37 ± 1.82 a	21.33 ± 1.28 a	9.07 ± 0.55 abc	20.45 ± 1.04 ab	11.03 ± 0.99 abc	19.09 ± 1.04 ab	14.07 ± 0.58 b	18.51 ± 0.59 a	16.33 ± 0.55 b	17.41 ± 1.01 a
Average		7.08 ± 3.41 A	19.46 ± 1.94 A	8.50 ± 1.43 A	18.50 ± 1.96 A	11.11 ± 2.23 A	17.49 ± 1.63 A	13.29 ± 2.84 A	16.85 ± 1.74 A	15.16 ± 3.21 A	15.96 ± 1.64 A
Source of variation											
N application rate (N)		ns	**	**	**	**	*	**	**	**	**
B addition rate (B)		ns	ns	ns	ns	ns	ns	ns	ns	*	ns
N × B		ns	ns	ns	*	ns	ns	**	*	**	ns

Different lowercase letters within the same column indicate significant differences among the treatments (*p* < 0.05); different capital letters indicate significant differences among the biochar (B) treatments; stars indicate the level of significance (* *p* < 0.05, ** *p* < 0.01), ns indicates not significant; B0—nil biochar; B1—4.2 t ha^−1^ year^−1^ of biochar; B2—8.4 t ha^−1^ 2 year^−1^ of biochar; N0—nil nitrogen; N1—180 kg Nha^−1^; N2—225 kg Nha^−1^; values are means ± SD (*n* = 3).

**Table 2:** The effect of different biochar treatments on the stem diameter coefficient (SDC) and cross-sectional moment of inertia (CSM) of the 1st to 5th internodes.

Treatment		1st		2nd		3rd		4th		5th	
		SDC	CSM/mm^4^	SDC	CSM/mm^4^	SDC	CSM/mm^4^	SDC	CSM/mm^4^	SDC	CSM/mm^4^
B0	N0	2.62 ± 0.15 a	5168.88 ± 551.29 b	4.28 ± 0.48 ab	4468.18 ± 181.60 c	5.29 ± 0.23 b	3604.19 ± 201.35 c	5.97 ± 0.45 b	2803.69 ± 100.81 c	7.55 ± 0.36 d	2061.36 ± 129.77 c
	N1	3.26 ± 0.96 a	10,011.91 ± 1323.43 a	3.90 ± 0.45 b	9517.46 ± 1899.01 a	5.74 ± 0.43 b	7225.52 ± 363.82 a	7.60 ± 0.53 b	5494.63 ± 849.91 ab	8.17 ± 0.48 cd	4503.18 ± 407.11 a
	N2	3.21 ± 0.25 a	7215.15 ± 1263.60 ab	5.21 ± 0.20 ab	5936.69 ± 575.96 bc	6.78 ± 0.65 ab	5263.79 ± 1685.54 abc	9.41 ± 0.76 a	3931.23 ± 402.97 bc	10.14 ± 0.63 ab	3793.91 ± 182.34 a
Average		3.03 ± 0.59 A	7465.31 ± 2312.15 A	4.46 ± 0.67 A	6640.78 ± 2460.07 A	5.93 ± 0.78 A	5364.50 ± 1793.90 A	7.66 ± 1.57 A	4076.52 ± 1262.27 A	8.62 ± 1.25 A	3452.81 ± 1112.37 A
B1	N0	1.99 ± 0.53 a	6627.97 ± 1203.33 ab	3.69 ± 0.77 b	5143.64 ± 1251.92 c	5.16 ± 0.77 b	4921.73 ± 2308.29 abc	6.47 ± 0.47 b	2940.60 ± 384.05 c	7.61 ± 0.63 d	2135.52 ± 432.63 c
	N1	2.35 ± 1.11 a	9632.24 ± 3444.52 a	4.48 ± 0.73 ab	6900.52 ± 992.78 abc	7.09 ± 1.67 ab	5292.08 ± 919.29 abc	7.51 ± 1.15 b	4722.03 ± 672.08 ab	9.20 ± 1.07 bcd	3994.15 ± 667.51 a
	N2	3.15 ± 1.02 a	7634.60 ± 252.25 ab	5.63 ± 0.50 a	6304.01 ± 1451.56 bc	6.51 ± 1.05 ab	5105.89 ± 501.06 abc	9.66 ± 0.31 a	4118.52 ± 652.17 bc	11.12 ± 0.39 a	3572.01 ± 649.94 ab
Average		2.50 ± 0.95 A	7964.94 ± 2257.83 A	4.60 ± 1.03 A	6116.06 ± 1328.01 A	6.25 ± 1.36 A	5106.57 ± 1277.42 A	7.88 ± 1.55 A	3927.05 ± 933.69 A	9.31 ± 1.65 A	3233.89 ± 987.84 A
B2	N0	2.82 ± 1.58 a	5220.46 ± 507.70 b	4.45 ± 1.42 ab	4179.71 ± 955.97 c	5.53 ± 1.13 b	3636.43 ± 740.77 c	6.33 ± 0.86 b	2925.46 ± 653.62 c	7.65 ± 0.94 d	2291.34 ± 586.40 bc
	N1	3.97 ± 2.34 a	6721.58 ± 3198.43 ab	5.02 ± 1.07 ab	5458.30 ± 2573.36 c	7.96 ± 1.80 a	4186.07 ± 1750.62 bc	9.85 ± 1.68 a	3836.99 ± 1977.18 bc	11.50 ± 1.98 a	3241.99 ± 1477.25 abc
	N2	2.45 ± 0.64 a	10,306.84 ± 2394.06 a	4.44 ± 0.30 ab	8674.79 ± 1721.86 ab	5.80 ± 0.71 b	6596.59 ± 1361.00 ab	7.61 ± 0.48 b	5786.80 ± 717.31 a	9.39 ± 0.27 bc	4563.38 ± 1009.72 a
Average		3.08 ± 1.60 A	7416.29 ± 3029.38 A	4.63 ± 0.94 A	6104.27 ± 2578.47 A	6.43 ± 1.61 A	4806.36 ± 1796.06 A	7.93 ± 1.83 A	4183.08 ± 1677.87 A	9.51 ± 2.00 A	3365.57 ± 1364.88 A
Source of variation											
N application rate (N)		ns	**	ns	**	*	*	**	**	**	**
B addition rate (B)		ns	ns	ns	ns	ns	ns	ns	ns	ns	ns
N × B		ns	ns	ns	*	ns	ns	**	*	**	ns

Different lowercase letters within the same column indicate significant differences among the treatments (*p* < 0.05); different capital letters indicate significant differences among the biochar (B) treatments; stars indicate the level of significance (* *p* < 0.05, ** *p* < 0.01), ns indicates not significant; B0—nil biochar; B1—4.2 t ha^−1^ year^−1^ of biochar; B2—8.4 t ha^−1^ 2 year^−1^ of biochar; N0—nil nitrogen; N1—180 kg Nha^−1^; N2—225 kg Nha^−1^; values are means ± SD (*n* = 3).

**Table 3:** The effect of different biochar treatments on maize plant height and internode dry weight.

Treatment		Dry Weight of Internode/g Plant^−1^						Plant
		1st	2nd	3rd	4th	5th	Other	Height/cm
B0	N0	2.77 ± 0.49 bc	3.10 ± 0.44 e	3.10 ± 0.46 f	2.93 ± 0.47 d	2.97 ± 0.23 c	13.07 ± 1.65 d	216.6 ± 12.0 e
	N1	5.10 ± 0.89 ab	6.20 ± 0.17 a	6.87 ± 0.50 a	7.10 ± 0.20 a	6.20 ± 0.44 ab	28.13 ± 2.02 b	252.7 ± 36.1 d
	N2	4.40 ± 0.78 abc	4.67 ± 0.25 cd	5.03 ± 0.55 cd	5.17 ± 0.35 c	5.07 ± 0.49 b	23.40 ± 3.01 c	294.8 ± 9.1 a
Average		4.09 ± 1.22 A	4.66 ± 1.37 A	5.00 ± 1.69 A	5.07 ± 1.83 A	4.74 ± 1.46 A	21.53 ± 6.96 B	254.7 ± 39.1 B
B1	N0	2.47 ± 0.40 c	3.37 ± 0.31 e	3.53 ± 0.38 ef	3.53 ± 0.38 d	3.30 ± 0.35 c	15.73 ± 2.07 d	227.2 ± 11.4 e
	N1	4.07 ± 1.12 abc	5.17 ± 0.25 bc	5.43 ± 0.42 bc	5.63 ± 0.45 bc	5.73 ± 0.35 ab	28.20 ± 2.55 b	283.1 ± 7.6 ab
	N2	5.10 ± 2.31 ab	5.80 ± 1.05 ab	5.83 ± 1.55 abc	6.60 ± 1.25 ab	6.40 ± 1.05 ab	28.60 ± 2.00 b	289.6 ± 7.0 ab
Average		3.88 ± 1.73 A	4.78 ± 1.23 A	4.93 ± 1.35 A	5.26 ± 1.52 A	5.14 ± 1.53 A	24.18 ± 6.62 A	266.6 ± 34.3 A
B2	N0	2.67 ± 0.84 c	3.83 ± 0.45 de	3.73 ± 0.51 def	3.50 ± 0.44 d	3.47 ± 0.61 c	15.67 ± 2.03 d	229.9 ± 9.9 de
	N1	2.87 ± 1.02 bc	4.00 ± 0.53 de	4.67 ± 0.50 cde	4.97 ± 0.81 c	5.10 ± 0.53 b	22.30 ± 4.42 c	277.9 ± 5.6 bc
	N2	6.10 ± 2.10 a	6.17 ± 0.93 a	6.53 ± 1.22 ab	6.87 ± 1.10 a	6.90 ± 1.48 a	33.80 ± 0.70 a	268.0 ± 9.8 c
Average		3.88 ± 2.08 A	4.67 ± 1.27 A	4.98 ± 1.42 A	5.11 ± 1.63 A	5.16 ± 1.71 A	23.92 ± 8.32 AB	258.6 ± 2.5 B
Source of variation								
N application rate (N)		**	**	**	**	**	**	**
B addition rate (B)		ns	ns	ns	ns	ns	ns	**
N × B		ns	**	**	**	*	**	**

Different lowercase letters within the same column indicate significant differences among the treatments (*p* < 0.05); different capital letters indicate significant differences among the biochar (B) treatments; stars indicate the level of significance (* *p* < 0.05, ** *p* < 0.01), ns indicates not significant; B0—nil biochar; B1—4.2 t ha^−1^ year^−1^ of biochar; B2—8.4 t ha^−1^ 2 year^−1^ of biochar; N0—nil nitrogen; N1—180 kg Nha^−1^; N2—225 kg Nha^−1^; Values are means ± SD (*n* = 3).

**Table 4:** Effect of different biochar treatments on maize yield and yield components.

Treatment		Yield (kg/ha)	1000-Grain Weight/g	Kernels per Ear	Ear Number (Ears/ha)
B0	N0	2416 ± 117 c	279 ± 10 d	260 ± 22 d	53,889 ± 4194 c
	N1	8880 ± 1033 b	326 ± 10 a	475 ± 28 b	60,000 ± 3333 bc
	N2	12,977 ± 1333 a	336 ± 7 a	574 ± 68 a	66,667 ± 4410 ab
Average		8091 ± 1333 A	314 ± 28 B	436 ± 144 A	60,185 ± 6532 A
B1	N0	3052 ± 441 c	294 ± 6 c	289 ± 42 cd	53,333 ± 6667 c
	N1	8915 ± 2340 b	325 ± 5 a	511 ± 17 b	66,111 ± 2546 ab
	N2	9307 ± 1008 b	327 ± 4 a	451 ± 25 b	70,000 ± 1667 a
Average		7091 ± 3298 A	315 ± 16 B	417 ± 103 A	63,148 ± 8393 A
B2	N0	2808 ± 1010 c	310 ± 5 b	212 ± 45 c	53,889 ± 5853 c
	N1	10,663 ± 614 b	334 ± 10 a	467 ± 15 b	67,778 ± 5853 ab
	N2	9515 ± 1260 b	330 ± 2 a	472 ± 11 b	65,556 ± 4811 ab
Average		7662 ± 3774 A	325 ± 13 A	384 ± 131 B	62,407 ± 8041 A
Source of variation					
N application rate (N)		**	**	**	**
B addition rate (B)		ns	*	*	ns
N × B		**	**	**	ns

Different lowercase letters within the same column indicate significant differences among the treatments (*p* < 0.05); different capital letter indicate significant differences among the biochar (B) treatments; stars indicate the level of significance (* *p* < 0.05, ** *p* < 0.01), ns indicated not significant; Values are means ± SD (*n* = 3); B0—Nil biochar; B1—4.2 t ha^−1^ year^−1^ of biochar; B2—8.4 t ha^−1^ 2 year^−1^ of biochar; N0—Nil nitrogen; N1—180 kg Nha^−1^; N2—225 kg Nha^−1^.

**Table 5:** The nine treatments of the experiment.

Levels	B0	B1	B2
N0	N0B0	N0B1	N0B2
N1	N1B0	N1B1	N1B2
N2	N2B0	N2B1	N1B2

B0—nil biochar; B1—4.2 t of biochar ha^−1^ year^−1^; B2—8.4 t of biochar ha^−1^ 2 year^−1^; N0—nil nitrogen; N1—180 kg Nha^−1^; N2—225 kg Nha^−1^.

## Data Availability

The data presented in this study are available on request from the corresponding author.
